# Analysis for interaction between interleukin-35 genes polymorphisms and risk factors on susceptibility to coronary heart disease in the Chinese Han population

**DOI:** 10.1186/s12872-020-01811-8

**Published:** 2021-01-06

**Authors:** Hu Li, Ying-Xue Liu, Jin-Yan Huang, Yu-Feng Zhu, Kui Wang

**Affiliations:** 1 Deputy Chief Physician, Director of Cardiovascular Department of the First Naval Hospital of Southern Theater Command, PLA, Haibin Avenue 10, Zhanjiang, 524005 Guangdong Province People’s Republic of China; 2 Out Patient Department of the First Naval Hospital of Southern Theater Command, PLA, Zhanjiang, 524005 People’s Republic of China; 3 Department of Cardiovascular of the First Naval Hospital of Southern Theater Command, PLA, Zhanjiang, 524005 People’s Republic of China

**Keywords:** Coronary heart disease, Single nucleotide polymorphisms, Interleukin-35, Interaction, Smoking

## Abstract

**Background:**

The relationship between IL-35 genes polymorphism and susceptibility to coronary heart disease has not been tested in the largest Han population in China. The aim of this study was to explore the effect of single nucleotide polymorphisms (SNPs) of interleukin-35 (*IL-35*) genes and its relationship with environment on the risk of coronary heart disease (CHD).

**Methods:**

We performed Hardy–Weinberg equilibrium test on the control group. The relationship between the four SNPs of *IL-35* genes and the risk of coronary heart disease was studied by multivariate logistic regression. The best interaction was identified with generalized multifactor dimensionality reduction (GMDR). Logistic regression was used for investigation on association between four SNPs and CHD risk.

**Results:**

Logistic regression analysis showed that the C allele of rs428253 and the G allele of rs2243115 were independently correlated with increased risk of CHD, and adjusted ORs (95% CI) were 1.91 (1.28–2.64) and 1.80 (1.30–2.23), respectively. However, there was no significant association between CHD and rs4740 or rs568408. GMDR model indicated a best model for CHD risk consisted of rs428253 and current smoking, which scored 10/10 for both the sign test and cross-validation consistency (*p* = 0.010). Therefore, this overall multi-dimensional model had the highest cross-validation consistency, regardless of how the data were divided. This provided an evidence of gene–environment interaction effects. We also found that current smokers with rs428253-GC/CC genotype have the highest CHD risk, compared to never smokers with rs428253-GG genotype, OR (95% CI) = 3.04 (1.71–4.41), after adjustment for age, gender, hypertension, T2DM and alcohol consumption status.

**Conclusions:**

In this study, the C allele of rs428253 and the G allele of rs2243115, and the interaction rs428253 and current smoking were correlated with increased risk of CHD.

## Background

In recent years, coronary heart disease (CHD) has become the main cause of the incidence rate and mortality rate in developed China and developing countries [1]. In recent years, the incidence and mortality rate of CHD in China has increased rapidly, and the age of onset is younger. In young people (below 40 years old), about 700,000 people die from CHD every year [2, 3]. The etiology of CHD is very complex, and risk of CHD is not only affected by conventional environmental factors, but also by genetic variation [4]. Previous study has reported many environmental risk factors related to coronary heart disease, including blood lipid concentration, blood pressure, smoking, diabetes and so on [5]. In addition, inflammation also was reported associations with atherosclerosis development, which was an important risk factor for CHD [6].

Previous studies have reported that some inflammatory cytokine gene polymorphisms are associated with the risk of CHD, including cytokines in the IL-12 family [7, 8]. IL-35 is an anti-inflammatory cytokine and a member of IL-12 family. It is a heterodimer composed of p35 (IL-12A) and EBI3 subunits [9, 10]. Previous studies have reported that this genetic variation is related to the susceptibility of Alzheimer's disease and Graves' disease [11, 12], while *EBI3* genetic variation may affect the risk of allergic rhinitis (AR) and tuberculosis in Chinese [13, 14]. Recently, only two studies [15, 16] have also studied the relationship between *IL-35* genes polymorphism and CHD susceptibility. As far as we know, the relationship between *IL-35* genes polymorphism and susceptibility to coronary heart disease has not been tested in the largest Han population in China. Lin et al. [16] performed a case–control study for Chinese population, but these participants were all Chinese Zhuang. In addition, CHD is a multifactorial disease caused by both genetic and environmental factors [4] and gene–environment interactions [17]. The aim of this study was to evaluate the influence of SNPs within *IL-35* gene and their interaction with environment on susceptibility to CHD.

## Methods

### Participants

The population of current study was composed of 921 CHD patients and 926 age- and gender-matched controls. All CHD patients were recruited from the First Naval Hospital of Southern Theater Command. CHD was defined according to the World Health Organization criteria [18]. Participants with the following diseases will be excluded from the study cohort: heart related diseases, autoimmune diseases, chronic inflammatory diseases. The control participants were all the subjects who came to our hospital for routine occupational physical examination and voluntarily participated in this study. And those with CHD, hypertension and history of CHD were excluded from the control group. All the subjects were Han people, and there was no genetic relationship between them. Informed consent was obtained from each participant. In the study, we collected general demographic data and physical examination data of the subjects by questionnaire survey and physical measurement. The questionnaire includes general demographic information, life-style, smoking and alcohol consumption. This study has been approved by ethics committee of the First Naval Hospital of Southern Theater Command.

### Extraction of genomic DNA and genotyping

We selected 4 SNPs according to the following criteria from dbSNP algorithm (http://www.ncbi.nlm.nih.gov/projects/SNP): firstly, the MAF > 5% in the database; secondly, the relationship between SNPs and CHD was not verified in the previous studies. Genomic DNA from whole blood containing EDTA was isolated strictly following the instructions of the manufacturer. Four SNPs including rs428253, rs4740, rs2243115 and rs568408 were selected. Genotyping for rs2243115 and rs568408 was performed using polymerase chain reaction (PCR) and following restriction fragment length polymorphism (RFLP). Genotyping for rs428253 and rs4740 using TaqMan genotyping assays on an ABI Prism 7900HT Fast Real-Time PCR System according to the manufacturer’s instructions (Applied Biosystems, Foster City, CA, USA). The primers and assays used for genotyping were listed in Table 1.

**Table 1:** Description of 4 SNPs and the assays or primers designed for genotyping

SNPs	Chromosome	Functional Consequence	Major/minor alleles	Genotyping assays or primers
*EBI3*	19:4229916	Intron variant	G > C	GAATTTGAGTCACACTCATTCCTTT[C/G]
rs428253				GTTTCTTTTTGGTTTTGTTTTTTGA
*EBI3*	19:4236999	Missense variant, coding sequence variant	G > A	TGTGCGGCCCCGAGCCAGGTACTAC[A/G]
rs4740				TCCAAGTGGCGGCTCAGGACCTCAC
*IL-12A* rs2243115	3:159988493	Upstream transcript variant, intron variant	T > G	Forward: 5′-AGAAAAGACCTGTGAACAAAACGACT-3′
				Reverse: 5′-AGATGGCTCACTAGATGCCAGG-3′
*IL-12A* rs568408	3:159995680	3 prime UTR variant, intron variant	G > A	Forward: 5′-GAAGGATGGGACYATTACATCCATAT-3′
				Reverse: 5′-CAGGATGGATATTTTCCCTTCT-3′

### Statistical analysis

Hardy Weinberg equilibrium (HWE) was tested using SNPstats (http://bioinfo.iconcologia.net/SNPstats). Chi-square test was used to compare the distribution of alleles, genotypes and categorical variables among groups. The mean ± standard deviation (SD) was used to represent the continuous variables of normal distribution, and Student t test was used to compare the differences between the two groups. Generalized multifactor dimensionality reduction (GMDR) [19] was used to determine the optimal interaction combination of four SNPs of *IL-35* genes and environmental risk factors. Logistic regression was used to analyze the relationship between four SNPs and the risk of CHD. When p value is less than 0.05, the statistical significance is significant.

## Results

In this study, a total of 1847 participants were enrolled, including 921 patients with CHD and 926 normal controls. Table 2 showed the different demographic characteristics in both case and control group. The mean age of all participants is 60.9 ± 10.8 years old. There are no statistically significant differences between case and control group in males, BMI and age (*p* > 0.05). Compared with control group, the mean of C-reactive protein (CRP) levels, the rates for hypertension and T2DM of case group are significantly different (all *p* < 0.05). In addition, the case group had higher alcohol consumption and smoking rates than control group.

**Table 2:** General characteristics of study participants in CHD patients and controls

Variables	CHD patients (n = 921)	Controls (n = 926)	*p*-values
Age (years) (means ± SD)	60.6 ± 11.3	61.3 ± 12.0	0.197
Males, N (%)	463 (50.3)	474 (51.2)	0.694
BMI (kg/m^2^) (means ± SD)	24.6 ± 9.1	23.9 ± 9.5	0.106
CRP (mg/l) (means ± SD)	32.2 ± 16.7	12.9 ± 7.5	< 0.0001
Hypertension, N (%)	389 (42.2)	272 (29.4)	0.000001
T2DM, N (%)	170 (18.5)	104 (11.2)	0.000013
Smoking status, N (%)			0.000012
Current	267 (29.0)	187 (20.2)	
Never	654 (71.0)	739 (79.8)	
Alcohol consumption, N (%)			0.000089
Current	319 (34.6)	243(26.2)	
Never	602 (65.4)	683 (73.8)	

*BMI* body mass index, *CRP* C-reactive protein, *T2DM* type 2 diabetic mellitus, *SD* standard deviation

All genotypes of the four SNPs follow by HWE distribution in the control group (all *p* values in HWE testing are greater than 0.05). The C allele frequency of rs428253 gene is 20.5% in the normal control group and 30.2% in patients with CHD. The minor (G allele) allele frequencies of rs2243115 gene are 26.3% in patients with CHD and 17.8% in normal controls. Logistic regression analysis showed that both the C allele of rs428253 and the G allele of rs2243115 were independently related to the increased risk of CHD. The adjusted OR (95% CI) was 1.91 (1.28–2.64) and 1.80 (1.30–2.23), respectively. However, there was no significant correlation between CHD and rs4740 or rs568408 (Table 3).

**Table 3:** Genetic risk evaluation of 4 SNPs within IL-35 gene and CHD risk

SNP	Genotypes or Alleles	Frequencies N (%)		Adjusted OR (95% CI)^a^	*p*-values
		CHD cases (N = 921)	Controls (N = 926)		
*EBI3-rs428253*					
	GG genotype	458 (49.7)	594 (64.1)	1.00 (ref)	
	GC genotype	369 (40.1)	285 (30.8)	1.87 (1.21–2.58)	< 0.001
	CC genotype	94 (10.2)	47 (5.1)	2.06 (1.45–2.75)	< 0.001
	G allele	1285 (69.8)	1473 (79.5)	1.00	
	C allele	557 (30.2)	379 (20.5)	1.91 (1.28–2.64)	< 0.001
*P* values for HWE			0.097		
*EBI3-rs4740*					
	GG genotype	483 (52.4)	543 (58.6)	1.00 (ref)	
	GA genotype	359 (39.0)	323 (34.9)	1.41 (0.95–1.97)	0.325
	AA genotype	79 (8.6)	60 (6.5)	1.56 (0.87–2.26)	0.561
	G allele	1325 (71.9)	1409 (76.1)	1.00	
	A allele	517 (28.1)	443 (23.9)	1.46 (0.92–2.04)	0.487
*P* values for HWE			0.205		
*IL-12A-rs2243115*					
	TT genotype	513 (55.7)	633 (68.4)	1.00 (ref)	
	TG genotype	332 (36.1)	257 (27.8)	1.76 (1.32–2.18)	< 0.001
	GG genotype	76 (8.3)	36 (3.9)	1.93 (1.23–2.65)	< 0.001
	T allele	1358 (73.7)	1523 (82.2)	1.00	
	G allele	484 (26.3)	329 (17.8)	1.80 (1.30–2.23)	< 0.001
*P* values for HWE			0.127		
*IL-12A-rs568408*					
	GG genotype	563 (61.1)	641 (69.2)	1.00 (ref)	
	GA genotype	312 (33.9)	253 (27.3)	1.31 (0.82–1.82)	0.436
	AA genotype	46 (5.0)	32 (3.5)	1.53 (0.74–2.33)	0.628
	G allele	1438 (78.1)	1535 (82.9)	1.00	
	A allele	404 (21.9)	317 (17.1)	1.37 (0.80–1.91)	0.541
*P* values for HWE			0.259		

^a^Adjusted for gender, age, status of smoking and alcohol consumption and BMI

**Fig. 1:**
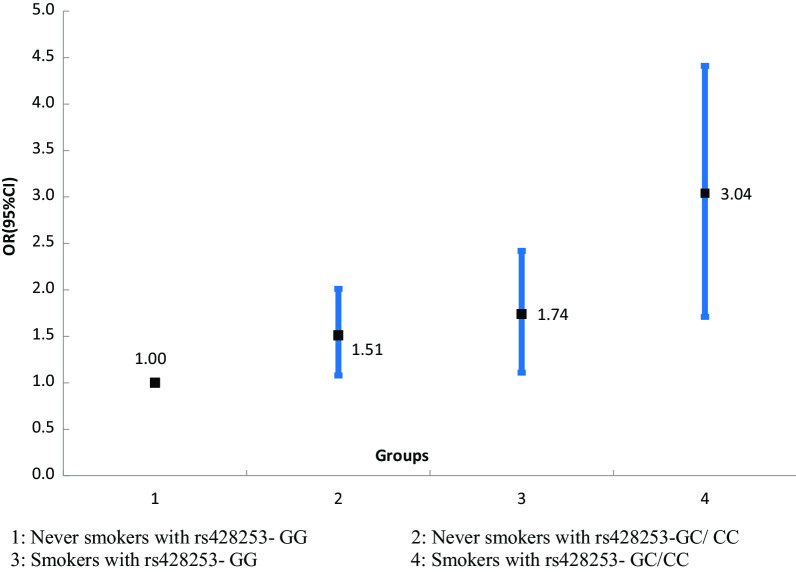
Stratified analysis for gene–smoking interaction on CHD risk using logistic regression

Further investigation on gene–gene and gene–environment interaction was tested using the GMDR methods, and the cross-validation consistency and testing accuracy were calculated. With covariate adjustments, the combination consist of rs428253 and current smoking is the best model, the cross-validation consistency of which was 10/10, and testing balanced accuracy of which was 0.632 (*p* = 0.010, Table 4). The fore-mentioned results provided an evidence for interaction effects between rs428253 and current smoking. But all gene–gene interaction combinations obtained from GMDR test have no statistical significance. We also conducted stratified analysis for interaction effect between rs428253 and current smoking using logistic regression. We found that current smokers with rs428253-GC/CC genotype have the highest CHD risk, compared to never smokers with rs428253-GG genotype, OR (95% CI) = 3.04 (1.71–4.41) (Fig. 1).

**Table 4:** GMDR analysis for the best interaction combination models

Locus no	Best combination	Cross-validation consistency	Testing balanced accuracy	*p*-values^a^
Gene–gene interactions^a^				
2	1, 3	7/10	0.607	0.321
3	1, 3, 2	6/10	0.532	0.528
4	1, 2, 4, 3	7/10	0.496	0.625
Gene–alcohol consumption interactions^b^				
2	1, 5	8/10	0.491	0.624
3	1, 2, 5	7/10	0.526	0.425
4	1, 2, 3, 5	5/10	0.518	0.746
5	1, 2, 3, 4, 5	6/10	0.521	0.857
Gene–smoking interactions^c^				
2	1, 6	10/10	0.632	0.010
3	1, 2, 6	7/10	0.532	0.172
4	1, 2, 3, 6	6/10	0.515	0.324
5	1, 2, 3, 4, 6	7/10	0.512	0.425

rs428253, rs4740, rs2243115, rs568408, current alcohol consumption and current smoking were symbolized as 1–6, respectively

^a^Adjusted for age, gender, BMI, hypertension, T2DM, smoking and alcohol consumption

^b^Adjusted for age, gender, BMI, hypertension, T2DM and smoking

^c^Adjusted for age, gender, BMI, hypertension, T2DM and alcohol consumption

## Discussion

The current study indicated that both the C allele of rs428253 and the G allele of rs2243115 were correlated with increased risk of CHD, but rs4740 or rs568408 were not statistically associated with CHD risk. The rs428253 belongs to *EBI3* gene, which located on chromosome 19p13.3. The rs2243115 belongs to *IL-12A* subunit, which is located on chromosome 3q25.33. Previously, several studies have investigated the association between the two SNPs and inflammatory diseases [11, 12]. So far, there are few studies about the effect of *IL-35* polymorphism on cardiovascular disease (CVD). Zhang et al. [14] confirmed that *EBI3*-rs428253 polymorphism is related to the chronic rhinosinusitis and allergic rhinitis risk reduction. So far, only two studies [15, 16] have confirmed the relationship between *IL-35* genes and CHD risk, but the conclusions of the two studies are inconsistent. A study [15] for Mexico populations suggested a relationship of the *EBI3* SNPs with IL-35 levels, and *EBI3*-rs428253 and *IL-12a*-rs2243115 gene polymorphisms play an important role in the mechanism of CHD risk reduction. However, a recent case–control study in the Chinese population [16] suggested a statistical correlation between the *EBI3* rs428253 mutation genotype and the risk of CHD, but there was no statistical significance between the mutation genotype of *IL-12A* rs2243115 and the risk of CHD. The study also found that there was no significant difference in the level of *IL-35* between different genotypes in the healthy control group. Although two case–control studies have been performed previously, but the two studies concluded inconsistent results, and just one study was performed for Chinese Zhuang population, but no study focused on Chinese Han population, which was the largest race in China. So, the different race may lead to different results. The biological mechanism of the association between *IL-35* genes and CHD susceptibility is not well established, previous studies suggested that EBI3-rs428253 may be involved in the modification of LEF1 binding site [15, 21], and play an important role in granulocyte proliferation and differentiation [22]. SNP-rs428253 in *EBI3* gene is related to the occurrence and development of CHD, which may be caused by regulating β—Catenin pathway and Treg pathway rather than by influencing the production of IL-35, because in the IL-35 levels was not different among different genotype of rs428253 in controls of study by Lin et al. [16]. In this study, we found that rs4740 and rs568408 was not associated with CHD risk, which was consistent with study by Lin et al. [16]. Although Posadas-Sánchez et al. [18] found that *IL-35 g*enes rs4740 and rs568408 were not associated with CHD risk in Mexicans, but the two SNPs genotypes were correlated with IL-35 levels in healthy Mexicans.

The pathogenesis of CHD is very complex, and is not only affected by genetic factors and environmental factors independently, but also by the synergistic effect between them [23, 24]. We were all known that alcohol consumption and smoking were two main modifiable risk factors for CHD [25, 26]. In this study, the smoking and alcohol consumption rates were higher in cases than controls, which indicated that smoking and alcohol consumption were two risk factors for CHD. So, we performed a GMDR analysis for gene–smoking or alcohol consumption interaction, and the results suggested a significant interaction between rs428253 and current smoking. Current smokers with rs428253—GC/CC genotype have the highest CHD risk, compared to never smokers with rs428253—GG genotype. Environmental factors can cause phenotypic differences by influencing gene expression regulation. Therefore, the study of *IL-35* gene–environment interaction is helpful for us to better understand the occurrence of CHD.

There were several limitations in this study. Firstly, the sample size is not large enough, so this is only a preliminary study of the polymorphism of this locus, the results obtained from current study need to be verified in the study with larger sample size and in different populations. Secondly, we just selected four SNPs in this study. In the future, we will investigate the relationship between multiple loci of *IL-35* gene and CHD, in order to better understand the mechanism of CHD from the perspective of genetics. Thirdly, the G allele frequency of rs2243115 was higher than that in gene database, so the selection bias may exist in this study. Lastly, we did not obtain the information on lipid gram data, such as total cholesterol, LDL or perhaps Lp (a) values, so these lipid values could not be adjusted in the regression models. IL-35 levels were not measured in the study subjects, so we could not observe the difference of IL-35 levels between cases and controls.

## Conclusions

The C allele of rs428253 and the G allele of rs2243115 are correlated with increased risk of CHD. We also find a significant interaction between rs428253 and current smoking associated with CHD risk. In addition, we also found the interaction between rs428253 and current smoking, which added more detailed mechanism for relationship between gene, environmental risk factors and CHD susceptibility.

## Data Availability

The datasets generated and/or analyzed during the current study are available from the corresponding author on reasonable request.

## Abbreviations

CHD: Coronary heart disease
SNP: Single nucleotide polymorphisms
IL-35: Interleukin 35
GMDR: Generalized multifactor dimensionality reduction
PCR-RFLP: Polymerase chain reaction—based restriction fragment length polymorphism
HWE: Hardy–Weinberg equilibrium
